# Stability of Nanometer-Thick Layered Gallium Chalcogenides and Improvements via Hydrogen Passivation

**DOI:** 10.1021/acsanm.3c03899

**Published:** 2023-10-26

**Authors:** Yael Gutiérrez, Stefano Dicorato, Elena Dilonardo, Fabio Palumbo, Maria M. Giangregorio, Maria Losurdo

**Affiliations:** † Istituto di Chimica della Materia Condensata e delle Tecnologie per l’Energia, ICMATE, CNR, C.so Stati Uniti 4, 35127 Padova, Italy; ‡ Physics Department, University of Oviedo, 33007 Oviedo, Spain; § Institute of Nanotechnology, CNR-NANOTEC, via Orabona 4, 70126 Bari, Italy

**Keywords:** 2D semiconducting layered materials, monochalcogenides, GaS, GaTe, GaSe, air stability

## Abstract

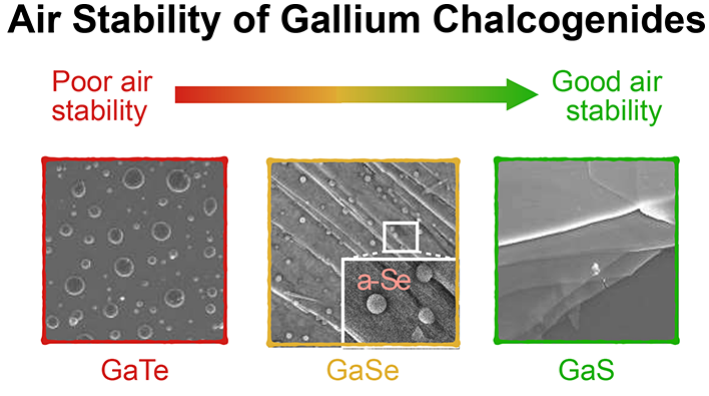

The gallium monochalcogenides family, comprising gallium sulfide (GaS), gallium selenide (GaSe), and gallium telluride (GaTe), is capturing attention for its applications in energy storage and production, catalysis, photonics, and optoelectronics. This interest originates from their properties, which include an optical bandgap larger than those of most common transition metal dichalcogenides, efficient light absorption, and significant carrier mobility. For any application, stability to air exposure is a fundamental requirement. Here, we perform a comparative study of the stability of layered GaS, GaSe, and GaTe nanometer-thick films down to a few layers with the goal of identifying the most suitable Ga chalcogenide for future integration in photonic and optoelectronic devices. Our study unveils a trend of decreasing air stability from sulfide to selenide and finally to telluride. Furthermore, we demonstrate a hydrogen passivation process to prevent the oxidation of GaSe with a higher feasibility and durability than other state-of-the-art passivation methods proposed in the literature.

## 1 Introduction

**Figure 1:**
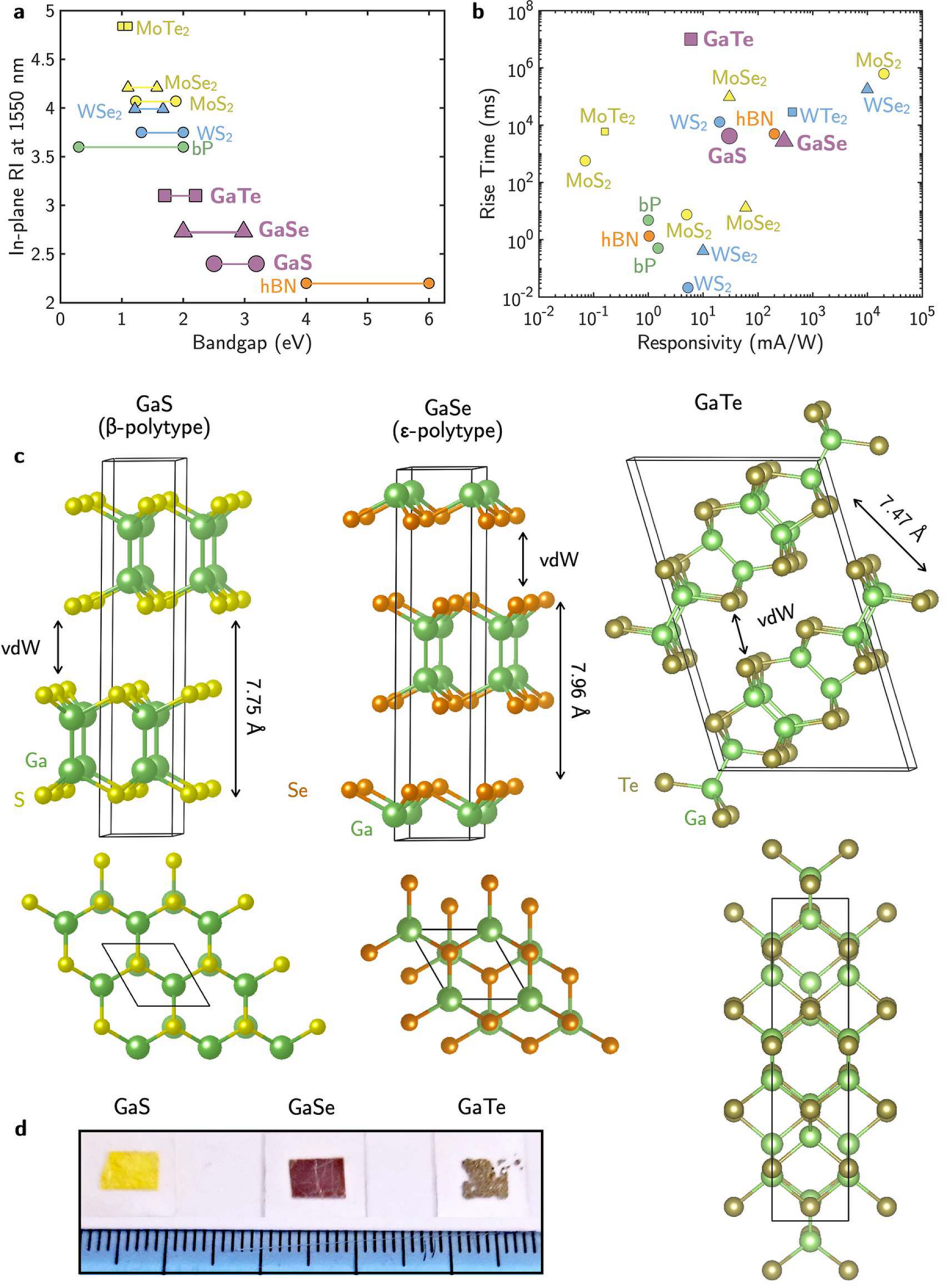
(a) In-plane real refractive index (RI) at the telecom wavelength of 1550 nm vs the optical bandgap of Ga monochalcogenides for the bulk and the monolayer^1,18,24,30−34^ compared to other 2D semiconductors.^35−38^ The optical bandgap of the bulk is on the left side of the bar, whereas that of the monolayer is indicated at the right of the bar. (b) Responsivity vs response time for Ga monochalcogenide-based photodetectors^8^ compared to other 2D semiconductor-based devices.^8−12^ The different points for the same materials are from different literature sources^8−12^ showing the wide scattering of data depending on material quality. (c) Lateral and top views of the Ga monochalcogenides GaS, GaSe, and GaTe unit cells. (d) Picture of the studied thick films (thickness ≈ 1 μm) of GaS, GaSe, and GaTe.

Gallium monochalcogenides, namely, gallium sulfide (GaS), gallium selenide (GaSe), and gallium telluride (GaTe), are van der Waals layered semiconductors with tunable bandgaps from the visible to the blue spectral region, efficient light absorption, and large carrier mobility that make them attractive for photonic and optoelectronic applications. Interestingly, the optical bandgap energy of Ga chalcogenides fills the range between transition metal dichalcogenides (TMDs) and the insulating hexagonal boron nitride (h-BN), as shown in Figure 1. Specifically, Figure 1a shows that GaS, GaSe, and GaTe cover a space in the map of optical bandgap versus refractive index at the telecom wavelength of 1550 nm not covered by other 2D materials. Other interesting properties can be observed in Figure 1a. For instance, moving from bulk materials and thick films to nanometer-thick films down to monolayers leads to an increase in the optical bandgap, showcasing the tunability of the optical bandgap by the number of layers. Moreover, focusing specifically on the Ga monochalcogenides, reducing the mass of the chalcogen atom, i.e., Te → Se → S, corresponds to an increase in the optical bandgap and to a decrease in the refractive index of the material.

In particular, the optical bandgap of GaS, GaSe, and GaTe ranges from 1.7 to 3.2 eV,^1^ making these materials suitable for optoelectronic applications such as visible–UV photodetectors.^2−7^ As an example of their functional properties, Figure 1b compares the responsivity versus rise time of Ga chalcogenide photodetectors to other 2D semiconductors, revealing that the photoresponsivity and rise time achieved by Ga monochalcogenides are comparable or larger than those of other TMDs.^8−12^ Additionally, given the wide bandgap of the Ga monochalcogenides, emerging applications include photocatalysis for water splitting,^1^ hydrogen evolution catalysis,^13^ and photoelectrochemical (PEC) reactions.^14,15^

The layered crystal structures of GaS, GaSe, and GaTe diffes from those of other 2D chalcogenides as they are based on covalently bonded X–Ga–Ga–X (X = S, Se, Te) tetralayers held together by van der Waals forces that allow their exfoliation down to the monolayer, as shown in Figure 1c. GaS and GaSe crystallize in a hexagonal structure with different staking of the layers within the unit cell.^16,17^ Specifically, GaS preferentially crystallizes in the β-polytype (*P*6_3_/*mmc*) and has an indirect bandgap of 2.5 eV that increases to 3.19 eV for the monolayer due to quantum confinement effects.^18^ GaS has already been integrated in a new generation of UV photodetectors with short time response,^2−4,19,20^ in field effect transitors,^21^ and applied to hydrogen evolution catalysis^13^ and second harmonic generation.^22^ Moreover, very recently, this material has been proposed as phase-change material for reconfigurable on-chip photonic components.^23^

GaSe crystallizes mainly in the ε-polytype (*P*6̅*m*2), and has an indirect bandgap of 2.12 eV,^24^ which has been predicted to increase to 2.98 eV for the monolayer.^1^ GaSe has been integrated in several optoelectronic devices such as photodetectors and phototransistors.^5,25−27^ Moreover, GaSe has strong nonlinear optical response that enables strong second and third harmonic generations.^28,29^

Differently from GaS and GaSe, GaTe crystallizes in a monoclinic structure (*C*2*/m* space group) with two-thirds of the Ga–Ga bonds perpendicular to the layer and one-third almost in the plane of the layer.^39^ Regarding the bandgap of GaTe, some controversy exists. Some DFT calculations and literature sources reported GaTe as an indirect bandgap semiconductor, where the peak in the valence band and the valley in the conduction band are very close.^1,40^ Other authors reported GaTe as a direct bandgap semiconductor.^32,34,41^ This discrepancy could be ascribed to the different phases in which GaTe can crystallize. Unlike monophasic GaS or GaSe, GaTe can crystallize in two different phases, i.e., the stable monoclinic phase studied in this work, and a metastable hexagonal phase with a tetralayer structure similar to that of GaS and GaSe.^42^ For the metastable hexagonal phase, several authors reported an indirect bandgap for both the bulk and the monolayer.^1,40^ The stable monoclinic phase studied here has been reported to have a direct bandgap.^7^ Bulk GaTe has a bandgap of ≈1.60 eV,^32,34,41^ increasing to 2.06 eV for the monolayer.^34^ GaTe has been applied in optoelectronics as a photodetector^43−46^ and exhibited a strong nonlinear response.^47^

A summary of several structural and electronic properties, as well as applications of the Ga chalcogenides, can be found in Table 1.

**Table 1:** Summary of Structural (Polytype and Space Group), Electronic (Bandgap and Carrier Mobility), and Applications of Different Ga Monochalcogenides

					
GaX	Polytype	Space group	Bandgap (eV); Bulk/ML	Carrier mobility (cm^2^ V^–1^ s^–1^); Bulk/ML	Applications
GaS	β	*P*6_3_/*mmc*	2.5^18^/3.19^1^	80^21^/0.1^21^	UV photodetectors,^2−4,19,20^ field effect transitors,^21^ hydrogen evolution catalysis,^13^ second harmonic generation,^22^ and reconfigurable photonics^23^
				*n*-type	
GaSe	ε	*P*6̅*m*2	2.12^24^/2.98^1^	215^21^/0.6^21^	Photodetectors and phototransistors,^5,25−27^ second and third harmonic generation^28,29^
				*p*-type	
GaTe	–	*C*2*/m*	1.62^34^/2.06^34^	25^48^/0.2^7^	Photodetectors,^43−46^ second and third harmonic generation^47^
				*p*-type	

Nevertheless, a challenge still to be addressed for Ga monochalcogenides is their stability in air.

Here, we report a comparative study of the air stability of GaS, GaSe, and GaTe for nanometer-thick films down to a few layers samples obtained by mechanical exfoliation. Our study indicates a decreasing the air stability as the chalcogen atom mass increases, i.e., air stability of GaTe < GaSe < GaS. We also demonstrate a process of passivation by atomic hydrogen to improve the air stability of GaS and GaSe.

## 2 Results

### 2.1 Gallium Sulfide

**Figure 2:**
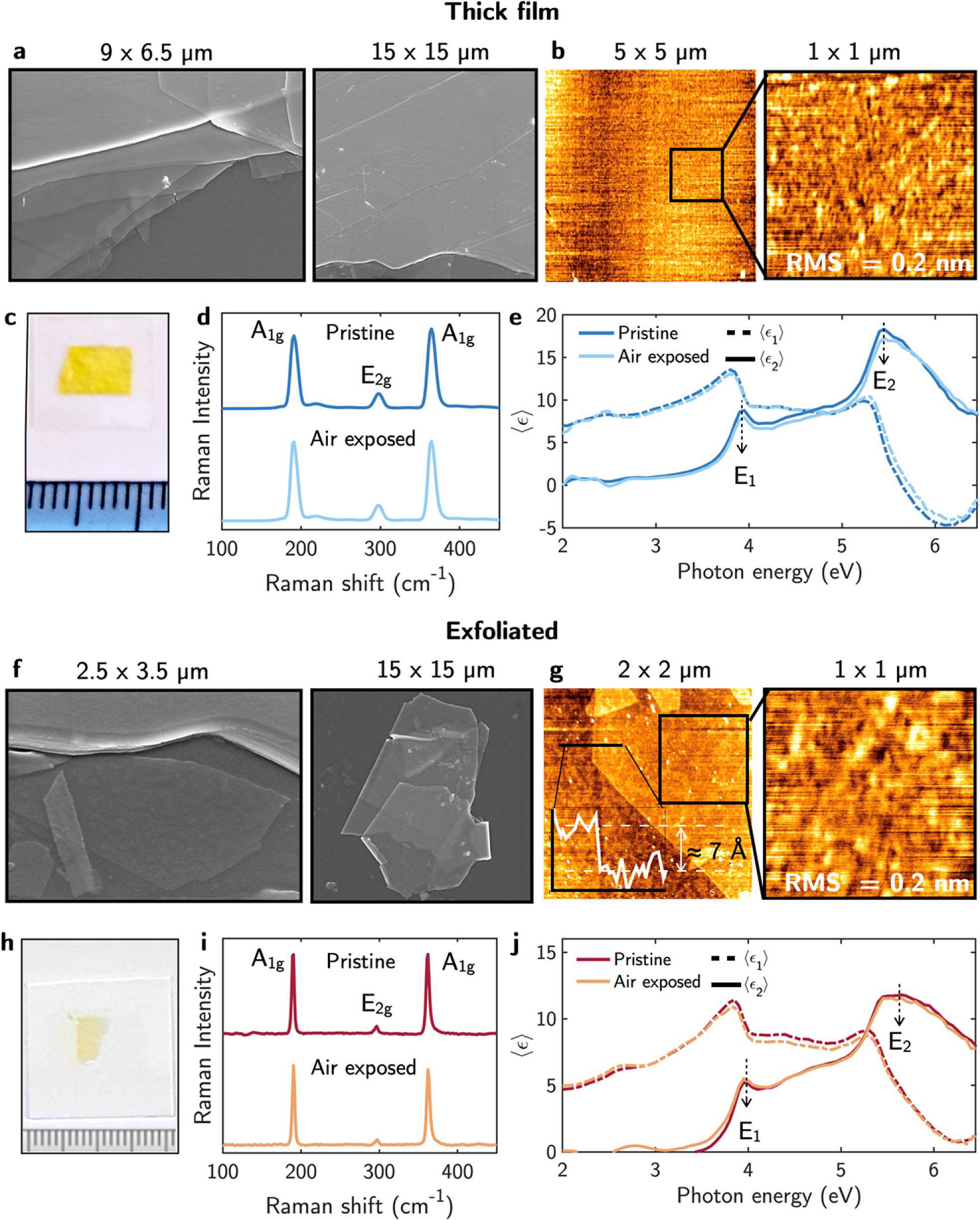
(a) SEM and (b) AFM topography of GaS thick film samples after air exposure. The measured root-mean-square roughness RMS is 0.20 ± 0.05 nm. (c) Picture of the GaS thick film sample. (d) Raman and (e) pseudodielectric function, ⟨ϵ⟩ = ⟨ϵ_1_⟩ + *i*⟨ϵ_2_⟩, of the GaS thick films before and after one moth of air exposure. (f) SEM and (g) AFM topography of the GaS exfoliated sample after air exposure; the RMS is 0.21 ± 0.05 nm. The height profile of the analyzed flake is shown in the inset. The small dots of impurities seen on the flakes in (e) and (f) are probably still residues of the glue of the thermal tape used for the exfoliation. (h) Picture of the mechanically exfoliated GaS sample. (i) Raman and (j) pseudodielectric functions of the exfoliated GaS before and after one month of air exposure.

The air stability of a 1 μm-thick GaS layer (see Figure 2c) and of the GaS few layer exfoliated sample (see Figure 2h) was studied over a period of one month. Figure 2a and b shows SEM and AFM topography images after air exposure of the GaS thick layer revealing a uniform flat morphology with a root-mean-square (RMS) surface roughness of 0.20 ± 0.05 nm after air exposure.

For the exfoliated sample, the SEM images in Figure 2f show a morphology consisting of a few layers from monolayer to three layer GaS supported on a glass substrate, with very smooth surfaces (RMS ≈ 0.20 ± 0.05 nm) after air exposure, as measured by AFM in Figure 2g. Specifically, Figure 2g shows the AFM topography of a GaS exfoliated monolayer as indicated by the height profile included as inset, revealing a thickness of ≈0.75 ± 0.05 nm consistent with a monolayer.^18^ Similar morphology and roughness were measured statistically on the fresh samples (therefore, this is not repeated in the figure). Consequently, no significant change in the RMS surface roughness and morphology was observed in both the thick film and monolayer after one month of air exposure.

Electronic and structural properties were studied by spectroscopic ellipsometry and Raman spectroscopy. The real, ⟨ϵ_1_⟩, and imaginary, ⟨ϵ_2_⟩, parts of the pseudodielectric function measured on the bulk and few layers samples before and after one month of air exposure are shown in Figure 2e and j. The ⟨ϵ_2_⟩ spectra are dominated by the two well-defined critical points (CPs) of GaS at 3.95 (*E*_1_) and 5.45 eV (*E*_2_).^18^ In semiconductors, it is well-known that the CP at higher energy, i.e., *E*_2_, because of the low penetration depth of light (i.e., about 5 nm), is very sensitive to surface oxidation and/or roughening causing its quenching. The spectra for the pristine and air exposed samples are almost coincident, revealing the good air stability of GaS. This is also supported by the Raman spectra taken on the pristine samples and after one month of air exposure, shown in Figure 2d and i. The Raman spectra of GaS, characterized by the three peaks at 185, 291, and 357 cm^–1^ assigned to the *A*_1*g*_, *E*_2*g*_, and *A*_1*g*_ Raman modes,^49^ do not change after air exposure, and do not show additional peaks due to a possible phase segregation and oxidation to Ga_2_O_3_. The larger full width at half-maximum of the GaS thick film could be attributed to a higher defect density present on the thick film due to imperfect stacking of layers.

### 2.2 Gallium Selenide

**Figure 3:**
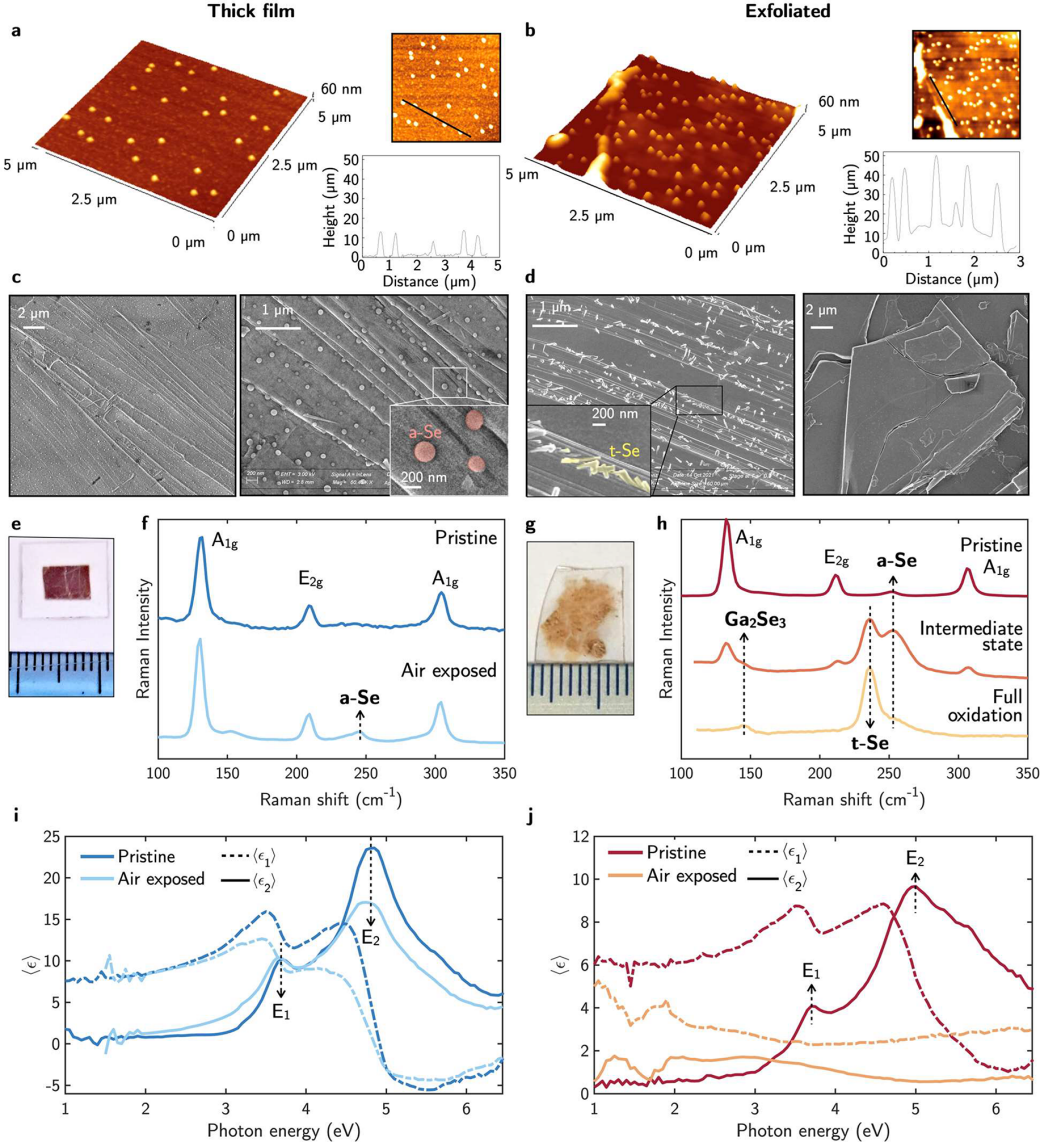
AFM 3D topography of the (a) GaSe thick film and (b) exfoliated sample after air exposure. The 2D topography maps indicate the region where the height profiles were taken. SEM image of the air exposed GaSe exfoliated sample showing different surface morphologies covered by (c) amorphous Se, *a-*Se, spherical particles and (d) trigonal Se, *t-*Se, nanorods. Pictures of the (e) thin film and (f) exfoliated samples. Raman spectra of the pristine and air exposed GaSe (g) thick film and (h) exfoliated sample. Pseudodielectric function, ⟨ϵ⟩ = ⟨ϵ_1_⟩ + *i*⟨ϵ_2_⟩, of the pristine and air exposed GaSe (i) thick film and (j) exfoliated sample.

Parallel experiments were run on a GaSe thick film (thickness ≈1 μm, see Figure 3e) and exfoliated few layers (see Figure 3g). The 3D AFM topography of the samples in Figure 3a and b measured after 1 week of air exposure shows surfaces partially covered by submicrometer particles, whose size and density increased in time. The height profile of the particles, also reported in Figure 3, shows that the particle diameter is ≈300 nm for both thick and exfoliated samples. Nevertheless, the height is higher for the exfoliated GaSe (≈ 40 nm) than the thick film GaSe (≈ 10 nm). The measured RMS surface roughnesses are 4 ± 1 nm for the thin film and 12 ± 2 nm for the exfoliated samples. The EDX compositional analysis of those particles indicated that they are Se rich. This is consistent with the similar morphological changes reported in transition metal diselenide HfSe_2_ flakes, which show similar spherical Se-rich particles appearing on their surface upon air exposure.^50^

Figure 3i and j shows the real, ⟨ϵ_1_⟩, and imaginary, ⟨ϵ_2_⟩, parts of the pseudodielectric function of the thick and the monolayer GaSe before and after air exposure. The ellipsometric spectra of the pristine thick film in Figure 3i shows good agreement with that reported in literature,^31^ with two main critical points in ⟨ϵ_2_⟩, i.e., *E*_1_ and *E*_2_ at 3.7 and 4.8 eV, respectively. After 1 week of air exposure, a broadening of both CPs and a quenching of the high energy CP was observed. This behavior is consistent with a roughening of the surface produced by the appearance of the Se submicrometer particles observed in the AFM topography. This phenomenon is further supported by the Raman spectra taken before and after air exposure and shown in Figure 3f. The pristine GaSe thick film presents the three *A*_1*g*_, *E*_2*g*_, and *A*_1*g*_ Raman modes at 136, 215, and 310 cm^–1^, respectively. After 1 week of air exposure, an additional peak appeared at 251 cm^–1^ due to the formation of amorphous selenium (*a*-Se) particles as also revealed by AFM. Noteworthy, after three months of air exposure, the Raman spectrum of the thick film preserved the *A*_1*g*_, *E*_2*g*_, and *A*_1*g*_ modes of GaSe demonstrating the self-limiting kinetics of the GaSe oxidation process to Se.

Conversely, the ⟨ϵ⟩ spectrum of the exfoliated GaSe in Figure 3j is completely quenched after 1 week of air exposure and no longer showed any CPs characteristic of GaSe. Correspondingly, the Raman spectra in Figure 3h measured just after the exfoliation, apart from the *A*_1*g*_^1^, *E*_2*g*_^1^, and *A*_1*g*_^2^ Raman modes at 136, 215, and 310 cm^–1^ characteristics of GaSe, already present a small component at 250 cm^–1^ attributed to *a*-Se. The oxidation process to *a*-Se in exfoliated few layers GaSe has the *a*-Se peak increasing in time along with the appearance of a new peak at 227 cm^–1^ compatible with trigonal Se (*t*-Se),^51^ and another component at 145 cm^–1^ due to to Ga_2_Se_3_. Further exposure to air leads to a Raman spectrum dominated by the main peak of *t*-Se at 227 cm^–1^ with a small contribution of two components at 250 cm^–1^ (*a*-Se) and 145 cm^–1^ (Ga_2_Se_3_). Therefore, during the oxidation process of GaSe by air exposure, *a*-Se appears at first and undergoes a phase change to crystalline *t*-Se. This transition is driven by the fact that *a*-Se comprises a distorted ring network, which has been demonstrated to be thermodynamically unstable.^51^ During the *a*-Se to *t*-Se transformation, the Se atoms form polymeric chains, which are the most stable modification.^51^ This is supported by the SEM image in Figure 3d of the fully oxidized flake covered by rod-like nanostructures compatible with a *t-*Se parallel organization of the Se polymeric chains.

The faster oxidation kinetics and larger *a*-Se particles for the exfoliated samples can be explained by the breaking of the van der Waals interaction between layers involving Se–Se atoms; consequently, the dangling bonds on the Se surface atoms enhance their tendency to interact among them, forming new polymeric Se–Se bonds. This aligns with the enthalpy and entropy arguments detailed in the subsequent discussion section.

### 2.3 Gallium Telluride

**Figure 4:**
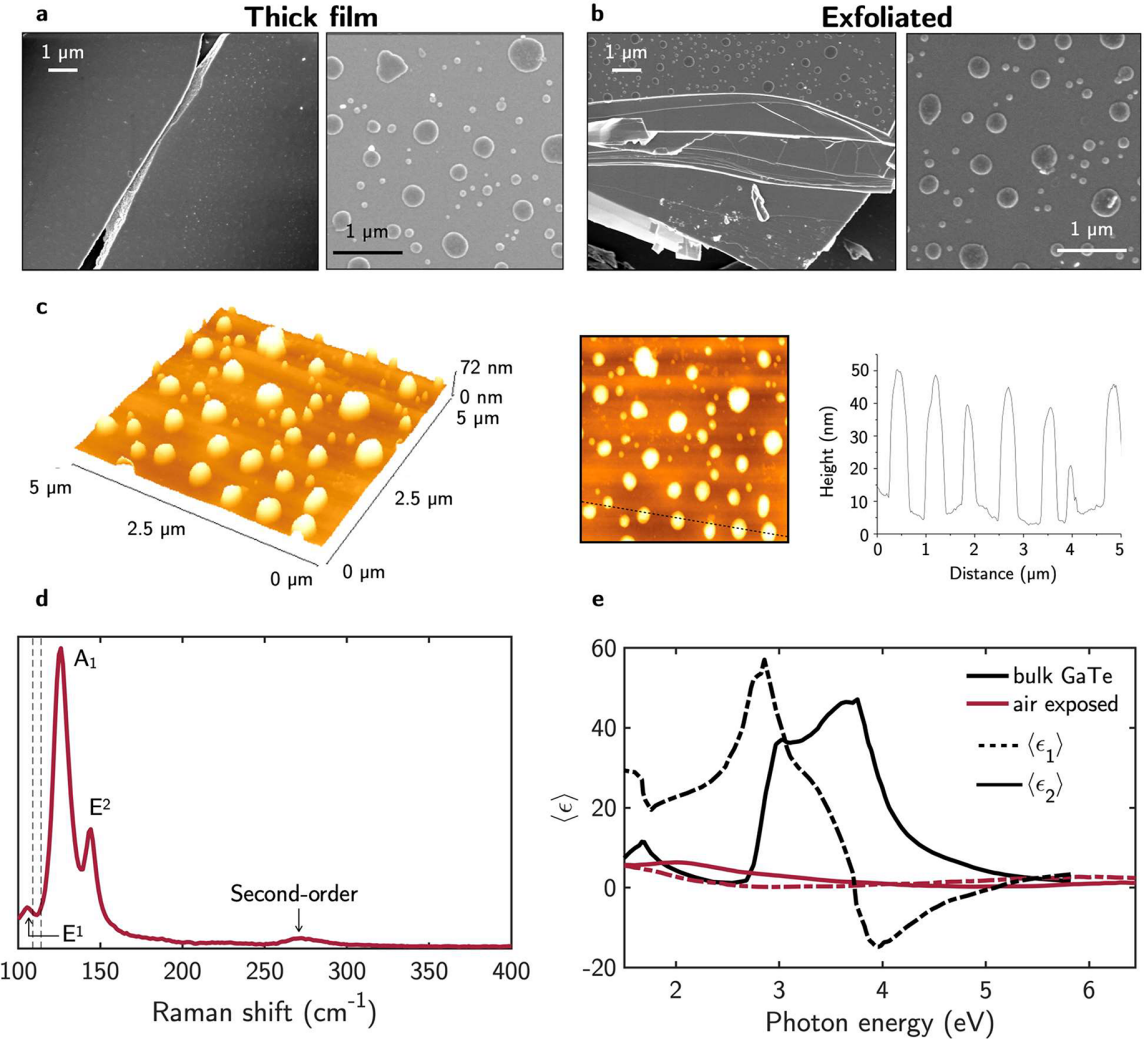
SEM images of the (a) thick film and (b) exfoliated sample. (c) AFM topography of the GaTe thick film after air exposure. (d) Raman of the GaTe thick film after air exposure. With dashed vertical lines are indicated the Raman modes of GaTe at 109 and 115 cm^–1^.^52^ (e) Pseudodielectric function, ⟨ϵ⟩ = ⟨ϵ_1_⟩ + *i*⟨ϵ_2_⟩, of air-exposed GaTe thick film. The dielectric function of bulk GaTe is shown in black.^55^

Rapid oxidation upon air exposure was observed for both the GaTe thick film and the exfoliated samples. As soon as the samples sealed in nitrogen were opened and characterized or exfoliated, complete oxidation was already evident. This was statistically observed on five different crystals. Thus, we were unable to measure the pristine state of the GaTe. SEM images in Figure 4a and b show the surface morphology of both thick film and exfoliated GaTe. In both cases, the surface presents droplets of a similar size and distribution. The AFM topography in Figure 4c shows that the droplets have a diameter ranging from 250 to 500 nm and a height in the range 30–50 nm. Those droplets are attributed to the segregation of Te onto the surface of GaTe upon air exposure. This is supported by the atomic concentrations measured by XPS, which samples a depth <10 nm, that revealed a Ga:Te ratio of 0.4. The RMS surface roughness of the air exposed samples is 15 ± 4 nm.

The Raman spectrum in Figure 4b shows two main bands at 126 and 144 cm^–1^ that differ from the most intense Raman modes of pristine GaTe, indicated with dashed lines, and that should appear at 109 and 115 cm^–1^.^52^ The observed Raman bands indicate oxidation of GaTe^52^ by the intercalation and chemisorption of oxygen.^53^ However, the observed Raman bands are not characteristic of TeO_2_ or GaTe-O_2_ but are the signature of the polycrystalline Te clusters forming at the surface as a result of Te segregation during the oxidation process.^54^ In particular, the peaks at 126 and 144 cm^–1^ are identified as the *A*_1_ and *E*^2^ Raman modes of trigonal Te. Additional peaks appearing in the Raman spectra with lower intensity at 105 and 270 cm^–1^ are assigned to the *E*^1^ and the second order Raman modes of trigonal Te.

Figure 4e shows the pseudodielectric function measured on the oxidized GaTe samples as compared to that of bulk GaTe dielectric function.^55^ The complete quenching of the critical points of the GaTe dielectric function supports the GaTe oxidation.

### 2.4 Chemical Changes of GaS, GaSe, and GaTe

The XPS semiquantitative analysis of the chemical elements on the surface of the investigated samples is summarized in Table 2. The percentages of the detected elements, including the unavoidable presence on the surface of contaminants such as carbon and oxygen, remain almost unchanged for pristine and aged GaS samples. These percentages remain reasonably consistent with the 1:1 stoichiometric ratio of GaS, signifying negligible surface alterations due to air exposure. This observation aligns coherently with all other collected data.

**Table 2:** XPS Surface Chemical Composition of Pristine and Aged GaX with X = S, Se, Te

	Pristine GaS	Aged GaS	Pristine GaSe	Aged GaSe	Aged GaTe
C (at.%)	36 ± 6	27 ± 7	60 ± 3	40 ± 2	64 ± 3
O (at.%)	24 ± 4	27 ± 8	11.1 ± 0.6	42 ± 2	30 ± 3
Ga (at.%)	19 ± 4	23 ± 3	14.2 ± 0.7	13.8 ± 0.7	1.4 ± 0.5
S (at.%)	19 ± 4	23 ± 6	–	–	–
Se (at.%)	–	–	14.6 ± 0.7	4.5 ± 0.2	–
Te (at.%)	–	–	–	–	4.5 ± 0.7
X/Ga	1.0 ± 0.4	1.0 ± 0.4	1.1 ± 0.1	0.33 ± 0.03	3.2 ± 1.6
VBM (eV)	1.8	1.70	1.2	1.0	0.05

During GaSe air aging, the selenium and oxygen atomic percentages vary considerably, with the Se/Ga atomic ratio decreasing from 1.1 to 0.33, implying a considerable surface modification of chemical composition and bond arrangements upon air exposure.

Conversely, GaTe reveals the immediate segregation of Te on the GaTe surface.

**Figure 5:**
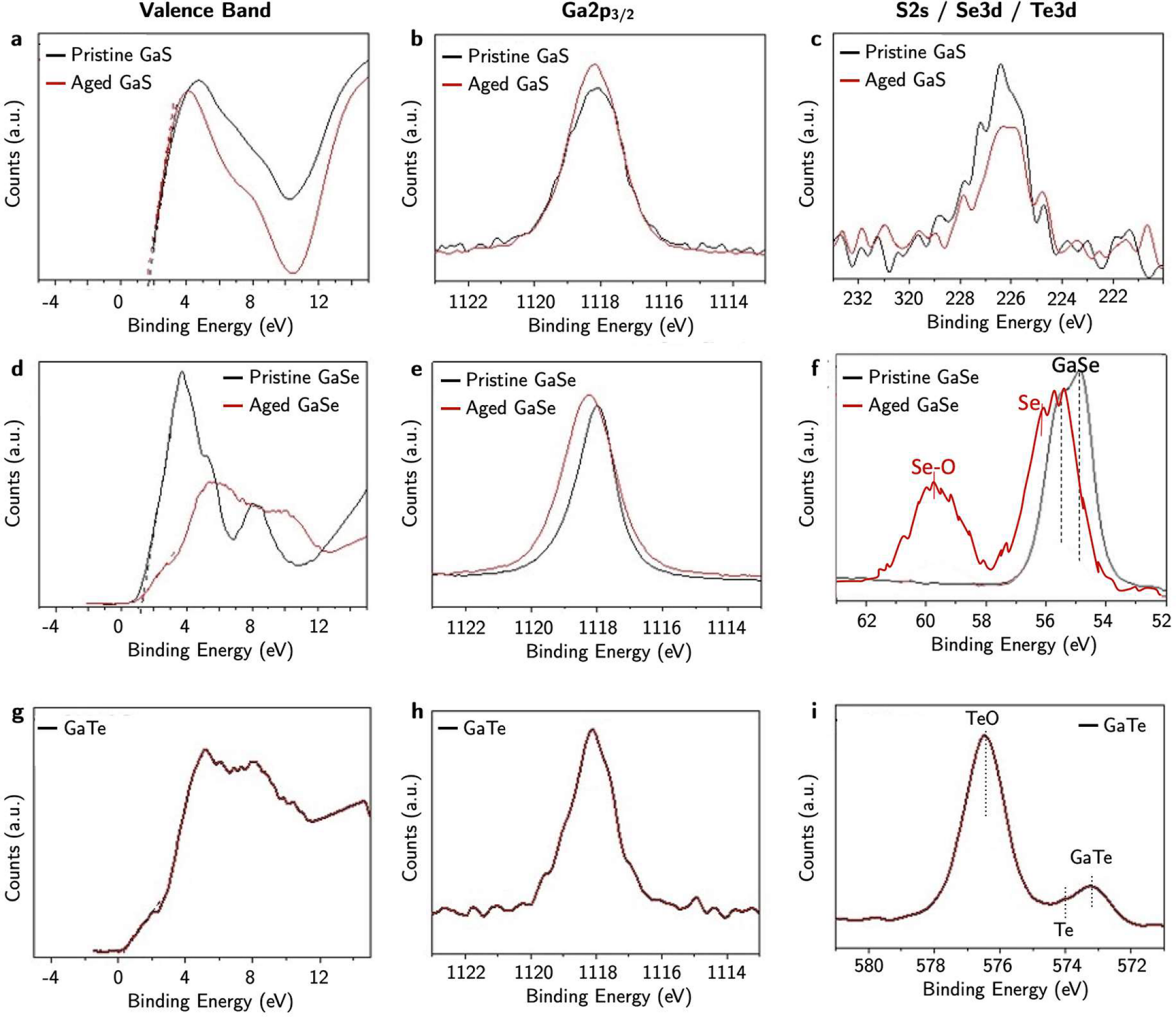
XPS (a) valence band, (b) Ga2*p*3/2,and (c) S2*s* core evels of pristine and aged exfoliated GaS. (d) Valence band, (e) Ga2*p*3/2,and (f) Se 3d core evels of pristine and aged GaSe. (g) Valence band, (h) Ga2*p*3/2,and (i) Te3*d* core evels of GaTe.

Figure 5 summarizes the main XPS core levels and valence band regions of the pristine and air exposed Ga chalcogenides. The XPS valence band in Figure 5a, d, and g describes the binding energy difference between the valence band maximum (VBM), determined by linear extrapolation of the valence band (VB) leading edge, and Fermi level (E_f_).^56^ The VBM values are also reported in Table 2. In GaS, the binding energy difference between VBM and *E*_f_ is 1.8–1.7 eV, demonstrating *p*-type behavior of GaS.^57^ In Figure 5b and c, the peak position and the full width at half-maximum (fwhm) of the high-resolution XPS Ga2*p*_3/2_ and S2*s* peaks of pristine and aged GaS are almost coincident and centered at 1118 and 226 eV, respectively, consistently with GaS.^58−60^

In pristine GaSe, the VBM is 1.2 eV, demonstrating also a GaSe *p*-type behavior;^57^Figure 5d shows that after air exposure, it red-shifted to 1.0 eV with the formation of a tail and an intensity decrease, because of surface oxidation.^52^ As observed in Figure 5e, the binding energy of Ga2*p*_3/2_ of GaSe increases from 1117.9 to 1118.2 eV upon air exposure, and the fwhm increases from 1.2 to 1.8 eV, indicating the contribution of oxides.^61^ Consistently, the Se3*d* peak shifts to higher binding energy with aging, with the appearance of an additional peak at 59.5 eV due to oxides, and also a shoulder peak at 55.6 eV due to elemental Se.^52^

The immediate surface oxidation of GaTe can be inferred by Figure 5g–i, as the Ga2*p*_3/2_ position at 1118.1 eV, and its fwhm of 1.6 eV can be interpreted as oxidized surface layer.^52,61^ The Te3*d* peak consists of the peak at 573.2 eV of GaTe, with a slight hump at 574.1 eV due to elemental Te, and a peak at 576.5 eV due to tellurium oxide.^52,62^

## 3 Discussion and Oxide Remediation

Ga chalcogenides show different degrees of air stability, with stability decreasing from GaS to GaSe and to the unstable GaTe. Specifically, the GaS stability in air has been checked over 8 months on a significant number of samples (>30) independently of their thickness. GaSe thick films undergo a self-limiting oxidation process, resulting in the surface segregation of *a*-Se and *t*-Se particles within a week. Finally, GaTe is very unstable in air and oxidizes after few hours of air exposure. This trend can be rationalized in terms of the decreasing reported values of cohesive energy, *E*_c,_ and of the enthalpy formation, Δf*H*°_298_, as well as increasing entropies *S*°_298_ (see in Table 2) when moving from S to Se and to Te chalcogen.^63^ Considering *E*_c_ and Δf*H*°_298_, a decrease in their values indicates a weaker bond between Ga and chalcogen atoms, thereby promoting oxidation. Therefore, the lower *E*_c_ and Δf*H*°_298_ are, the larger is the oxidation. An increase in entropy implies larger disorder or randomness in the system, and higher entropy can facilitate breaking bonds and mixing atoms, promoting oxidation reactions. Furthermore, the Ga chalcogen bond energies decrease down the period as demonstrated in Table 3.^39^ Lower bond energies correspond to a higher tendency of Ga chalcogen bonds to undergo oxidation.

**Table 3:** Cohesive Energy (*E*_c_), Enthalpy Formation Δ_f_*H*°_298_, and Entropy *S*°_298_ of GaS, GaSe, and GaTe as Reported in Ref (63) and Thermochemical Bond Energy as Reported by Ref (39)

	*E*_c_ (kJ mol^–1^)	Δ_f_*H*°_298_ (kJ mol^–1^) Theoretical/Experimental	*S*°_298_ (kJ mol^–1^ K^–1^)	Thermochemical bond energy (kJ mol^–1^)
GaS	–682	–135/–210 ± 10	57.1 ± 0.2	318
GaSe	–625	–116/–159 ± 10	71.7 ± 0.2	274
GaTe	–560	–79/–123 ± 4	80.7 ± 0.3	251

For GaSe, the main reactions of oxidation can be written as^52^
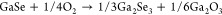
1
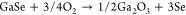
2

In the case of reaction 1, a further oxidation takes place:^61^
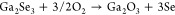
3bringing elemental selenium. This elemental Se at the surface aggregates forming particles as shown in Figure 3.

**Figure 6:**
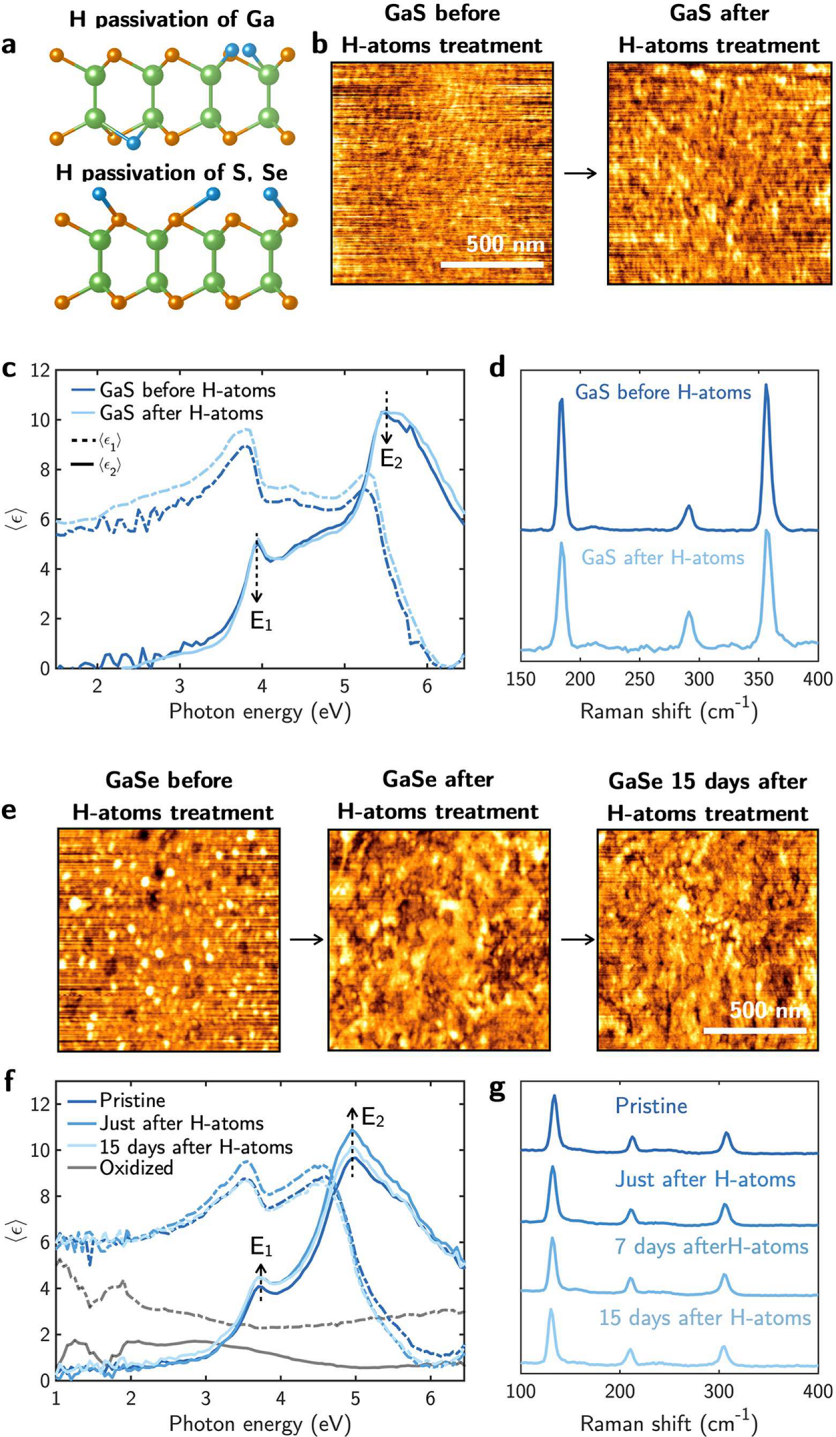
(a) Scheme of the crystalline structure of H-passivated GaS and GaSe. (b) AFM topography of aged GaS before and after a H-atoms treatment. (c) Pseudodielectric function, ⟨ϵ⟩ = ⟨ϵ_1_⟩ + *i*⟨ϵ_2_⟩, and (d) Raman spectra of GaS before and after a H-atoms treatment. (e) AFM topography of aged GaSe before, right after, and 15 days after a H-atoms treatment. (f) Pseudodielectric function, ⟨ϵ⟩ = ⟨ϵ_1_⟩ + *i*⟨ϵ_2_⟩, and (g) Raman spectra of GaSe pristine and right after, 7 days after, and 15 days after a H-atoms passivation treatment. In (f) is shown the ⟨ϵ⟩ spectrum of oxidized GaSe as reference.

In order to prevent oxidation of GaSe, several techniques have been reported in the literature, including N^+^ ion implantation^64^ and encapsulation by hexagonal boron nitride^65,66^ and PMMA.^67^ Here, we developed a room temperature soft hydrogen plasma passivation process as a new way to prevent the oxidation of GaSe flakes. Hydrogen passivation is an extended practice in semiconductors by which hydrogen atoms, H-atoms, are used to stabilize reactive dangling bonds that cause electronically active states that could enhance reactivity to air. This process is schematized in Figure 6a for both GaS and GaSe. Figure 6e shows the AFM topography of the GaSe exfoliated sample before and up to 15 days after H-plasma treatment. Before the plasma, i.e., after exfoliation, a small density of Se particles is already present on the surface of the sample. These are removed by the exposure to the H-atoms according to the reaction
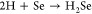
4with H_2_Se being a volatile compound with a boiling temperature of 232 K.^68^

No *a-Se* or *t-Se* particles were observed on the surface of the GaSe sample after 15 days of hydrogen cleaning and passivation. The sample maintained its clean surface, which was achieved through H-plasma treatment, indicating the successful blocking of the Se dangling bonds by H-atoms inhibiting the Se polymerization in clusters or nanorods and, consequently, inhibiting the oxidation process. This is also demonstrated by the structural and optical properties as Figure 6f shows that the pseudodielectric function ⟨ϵ⟩ of GaSe experiences an increase in the amplitude of the *E*_1_ and *E*_2_ critical points characteristics of GaSe after H-atoms treatment.

Furthermore, the ⟨ϵ⟩ spectra measured after 15 days of air exposure of the H-atoms passivated GaSe, unlike in the case of the untreated sample (oxidized), preserve the critical points characteristic of the material, indicating an increased stability to air exposure. This is also supported by the Raman analysis in Figure 6g, as spectra obtained after H-passivation exhibited no differences compared to those of pristine GaSe, without any peaks associated with either *a*-Se or *t*-Se appearing in time. Thus, the passivation of GaSe flakes by H-atoms has proven to be effective in inhibiting GaSe oxidation.

Noteworthy, the hydrogen passivation offers longer period of stability than PMMA encapsulation^67^ reported to be effective only up to 6 days. After this time, it was reported that the intensity of the *A*_1*g*_^1^ was significantly decreased with the *a*-Se band starting to appear after 5 days of exposure to air. The encapsulation of GaSe by h-BN has been reported to be more efficient.^66^ Nevertheless, given the state-of-the art for the production of large areas and transfer of this material, h-BN encapsulation might not be a cost effective and practical process for the encapsulation of mass production and large areas of monochalcogenides.

We also applied the H-atom passivation treatment to GaS even though it was already quite stable. In this case, the AFM morphology in Figure 6b as well as the optical and structural properties in Figure 6c and d indicate that there was no loss of sulfur by such room temperature treatment (by potentially 2H + GaS → H_2_S + Ga), but rather there was cleaning on the surface by removal of residual oxygen and carbon contaminants (shown by XPS in Table 2) and a slight smoothening of the surface with the RMS decreasing from 0.24 to 0.20 nm, consistent with the sharpening of the critical points in the pseudodielectric function. In the case of GaTe, although we tried to apply it, the crystals we had already showed a large Te segregation to recover GaTe.

## 4 Conclusions

In conclusion, we compared the stability in air of nanometer-thick films down to few layer exfoliated GaS, GaSe, and GaTe, identifying the most suitable Ga chalcogenide for future integration in nanophotonic and optoelectronic devices. Our study revealed a decrease in stability from GaS to GaSe to unstable GaTe. Optical, structural, morphological, and compositional properties indicated that GaS is remarkably stable, while GaSe oxidizes in a few days leading to segregation on the surface of micrometer-sized spherical and rod-like nanoparticles of *a-*Se and *t-*Se. GaTe results in surface segregation of polycrystalline Te after air exposure for a few hours of air exposure. Furthermore, we demonstrated that GaSe oxidation can be avoided by a hydrogen passivation process performed by exposure of the GaSe flakes to hydrogen atoms. Therefore, the hydrogen passivation process proposed here, already integrated in the semiconductor industry, could be a viable and reliable approach to passivate large area 2D materials.

## 5 Methods

### Sample Fabrication

Commercial bulk GaS, GaSe, and GaTe crystals (2D semiconductors and HQ graphene) were mechanically exfoliated using the standard tape methods. Corning glass substrates were treated overnight in hydrogen peroxide and then cleaned with isopropyl alcohol, acetone, and ethanol. Further details about the exfoliation process and exfoliated layers can be found in refs (20 and 69).

The stability of the materials was investigated at ambient conditions, meaning a temperature of ≈20 °C and a mean air humidity of ≈70%.

For each chalcogenide, at least 50 exfoliated samples were prepared from two commercial crystals and analyzed.

### Hydrogen Plasma Passivation

Remote hydrogen plasma was produced at 0.5 Torr with a H_2_ flow rate of 500 sccm by a radiofrequency (r.f.) 13.56 MHz at the low power of 80 W. Under those conditions, we verified that only neutral hydrogen atoms interact with the sample, avoiding ion or electron bombardment of the surface.^70^

### Layers Characterization

Raman and ellipsometric spectra and AFM scans were measured at least in five points for each sample.

Raman spectroscopy (LabRam Horiba) with a 532 nm wavelength laser (2 mW) was performed.

Morphology and surface roughness analysis were performed by atomic force microscopy (AFM) (AutoProbe CP, ThermoMicroscope).

Optical properties, namely, spectra of the pseudodielectric function, ⟨ϵ⟩ = ⟨ϵ_1_⟩ + *i*⟨ϵ_2_⟩, were measured by spectroscopic ellipsometry (UVISEL Horiba) in the photon energy range 0.75–6.5 eV with a resolution of 0.01 eV. The angle of incidence was set at 70°.

The elemental composition was evaluated by X-ray photoelectron spectroscopy (XPS). XPS analyses were performed by a scanning XPS microprobe (PHI 5000 Versa Probe II, Physical Electronics) equipped with a monochromatic Al Kα X-ray source (1486.6 eV) with a spot size of 200 mm. Survey (0–1200 eV) and high-resolution (HR) spectra (C 1s, Ga 2p_3/2_, S 2s, Se 3d, Te 3d) were acquired in FAT mode at a pass energy of 117.40 and 29.35 eV, respectively. Spectra were acquired at a takeoff angle of 45° with respect to the sample surface. Surface charging was compensated using a dual beam charge neutralization system, and the hydrocarbon component of the C 1s spectrum was used as internal standard for charging correction. It was fixed at 285.00 eV. The areas of the peaks were computed after fitting of the experimental spectra to Gaussian/Lorentzian curves and removal of the background (Shirley function). Surface atomic ratios were calculated from the peak area ratios normalized by the corresponding atomic sensitivity factors.

Scanning electron microscopy (SEM) was carried out for the morphological characterization of the samples with a Zeiss Supra 40 FEG SEM instrument equipped with a Gemini field emission gun. Analyses were carried out at an extraction voltage of 3 kV and a 30 μm aperture.
